# Mr. Cam, on the Use of the Trephine

**Published:** 1799-06

**Authors:** Thomas Cam

**Affiliations:** Surgeon to the Infirmary at Hereford. Hereford


### *To the Editors of the Medical and Physical Journal.*

GENTLEMEN,

I Have taken the liberty of enclosing a case which came under my care a few months ago, in which the trephine was used to great advantage.

It was a detachment of the dura mater from the cranium with extravasation, occasioned by a fall.

If you think it worthy of a place in your monthly publication, the insertion of it will much oblige,

GENTLEMEN,

Your very humble Servant,

THOMAS CAM, Jun.

*Hereford, April 21st,* Surgeon to the Infirmary at Hereford.

1799.

I was

I was fent for to a boy of about twelve years of age, upon the fourth of June laft, who, as he was leading a colt to the blackfmith's, met in the road a one-horfe chaife, which it ftartled at, and immediately fet off, the boy having the halter twifted feveral times round his arm, was thrown down with great violence, and dragged a confiderable diftance before he was difengaged. In an hour after the accident I faw the lad, whom I found apparently in a dying ftate; he was extremely cold and languid, his pulfe hardly perceptible, and his refpiration much oppreffed: I with much difficulty opened his mouth, and poured down fome wine, which after being repeated four or five times, feemed to revive him; and when he was a little recovered from his exceffive langour, I began to examine the injured parts. The firft thing that prefented itfelf was a fmall wound above the os frontis, with a mere detachment of the fcalp from the pericranium two inches in length, but no fracture or depreffion: the right clavicle was broken, and his back, arms, and legs, very much bruifed. His head having fuffered materially from the concuffion, I opened the temporal artery, but as the quantity of blood taken away, I judged not fufficient, I bled him in the arm. After this treatment he breathed with more eafe, and I left him much better than I could poffibly have expected. In the evening I faw him again, and as he was much heated, with a full pulfe, and great reftleffnefs, I took more blood from him. The next day he was quite as reftlefs, but his heat had nearly left him; fome aperient medicines were given, and with the affiftance of glyfters, evacuations were procured. On the third day he was with difficulty kept in bed, and could not be prevailed on to take the leaft nourifhment. On the fourth day, finding him not relieved, I removed the whole of the detached fcalp, which appeared a little difcoloured, but no difcovery of the nature of the injury could be made. On the fifth day, not being any better, his pulfe irregular, and his fæces and urine paffing involuntarily, I applied the trephine. When the cranium was elevated, a fanious fluid was difcharged, the dura mater was feparated from it, and the veffels turgid. On the next day after the operation, which was the fixth, he remained juft in the fame ftate, and very delirious. When the dreffings were removed, a confiderable quantity of the fame kind of fluid had made its efcape, and all the former fymptoms continued. On the eighth day there was very little difcharge, the cerebrum entirely filled up the opening in the cranium. Upon preffing the dura mater with my finger, more of the fanious fluid rufhed from the lower part of the frontal bone. On the ninth day obferving no amendment, I ufed the trephine again, to make a more depending outlet for the difcharge, the dura mater was covered with a dark flough, and as the difeafe extended further, I repeated the operation in two more places, to put me in poffeffion

 of of all the injured parts. On the tenth and eleventh days he was not much improved in his health, but upon the twelfth, he became more calm, evidently ſhewed ſigns of returning reaſon, took his food, and was compoſed. On the thirteenth, full as well as the day before. On the fourteenth day, ſtill improving, and upon the fifteenth the parts were covered with a well digeſted pus. On the ſixteenth and ſeventeenth days the ſore put on a very healthy appearance, and he took the cortex with apparent advantage. On the twenty-firſt day he was brought to Hereford for the convenience of attendance, a diſtance of four miles. At the end of two months, he returned into the country, and walked backward and forward to be dreſſed, the wound was then contracted to a very ſmall ſize, but prevented from quite healing, by ſome trifling exfoliations. The boy is perfectly recovered, and though weakly before the accident, now enjoys a good ſhare of health.

